# Inhibition of JNK-Mediated Autophagy Promotes Proscillaridin A- Induced Apoptosis *via* ROS Generation, Intracellular Ca^+2^ Oscillation and Inhibiting STAT3 Signaling in Breast Cancer Cells

**DOI:** 10.3389/fphar.2020.01055

**Published:** 2020-09-04

**Authors:** Muhammad Zubair Saleem, Mohammed Alshwmi, He Zhang, Syed Riaz Ud Din, Muhammad Azhar Nisar, Muhammad Khan, Shahid Alam, Gulzar Alam, Lingling Jin, Tonghui Ma

**Affiliations:** ^1^ College of Basic Medical Sciences, Dalian Medical University, Dalian, China; ^2^ Department of Clinical Laboratory, The First Affiliated Hospital of Dalian Medical University, Dalian Medical University, Dalian, China; ^3^ Department of Zoology, University of the Punjab, Lahore, Pakistan; ^4^ Department of Anatomy, Dalian Medical University, Dalian, China; ^5^ Advanced Institute for Medical Sciences, Dalian Medical University, Dalian, China; ^6^ School of Medicine and Holistic Integrative Medicine, Nanjing University of Chinese Medicine, Nanjing, China

**Keywords:** proscillaridin A, apoptosis, autophagy, signal transducer and activator of transcription 3 (STAT3), c-Jun N-terminal kinase, breast cancer

## Abstract

Breast cancer is the most heterogenous cancer type among women across the world. Despite concerted efforts, breast cancer management is still unsatisfactory. Interplay between apoptosis and autophagy is an imperative factor in categorizing therapeutics for cancer treatment. Proscillaridin A (PSD-A), a well-known cardiac glycoside used for cardiac arrest and arrythmias, has been unveiled in many cancer types but the underlying mechanism for apoptosis and autophagy in breast cancer is not fully understood. In our study, PSD-A restricted cell growth, inhibited STAT3 activation and induced apoptosis and autophagy in breast cancer cells *via* ROS generation and Ca^+2^ oscillation. Pretreatment of NAC and BAPTA-AM restored PSD-A induced cellular events in breast cancer cells. PSD-A induced apoptosis *via* DNA fragmentation, caspase-cascade activation, PARP cleavage, mitochondrial dysfunction, Bax/Bcl-2 proteins modulation and ER chaperone GRP78 inhibition along with decreased phosphorylation of ERK1/2. Inhibition of STAT3 activation was found to be associated with decreased phosphorylation of SRC. Moreover, PSD-A induced events of autophagy i.e. conversion of LC3-I to LC3-II, and Atg3 expression *via* JNK activation and decreased mTOR and AKT phosphorylation. In this study, pretreatment of SP600125, a JNK inhibitor, reduced autophagy and enhanced STAT3 inhibition and apoptosis. Additionally, SB203580, a commercial p38 inhibitor, stimulated STAT3 activation and improved autophagic events rate in breast cancer cells, displaying the role of the MAPK signaling pathway in interplay between apoptosis and autophagy. Our data suggest that the rate of apoptotic cell death is improved by blocking JNK-induced autophagy in PSD-A treated MCF-7 and MDA-MB-231 breast cancer cells.

## Introduction

Breast cancer is the most prevalent heterogeneous type of disease with a high incidence and mortality rate. It is the second leading cause of cancer-related death in women after lung cancer (Yuan et al., 2017). In the United States, 246,660 new cases (29% of all cancer cases) were estimated among women in 2016 (Siegel et al., 2016). According to American Cancer Society Cancer Facts and Figures 2018, 266,120 new invasive breast cancer cases among women and over 2,550 cases in men are projected to be diagnosed that speculate approximately 40,920 women’s and 480 men’s deaths in 2018 (Sharma and Kumar, 2018).

Breast cancer has been classified into different molecular subtypes, specifically, based on presence or absence of hormone receptors (HR^+^/HR^-^) for estrogen or progesterone and excessive level of human epidermal growth factor receptor 2 (HER2^+^/HER2^-^). Furthermore, Luminal A (HR^+^/HER2^-^) and Luminal B (HR^+^/HER2^+^) are hormone receptor positive breast cancer subtypes, accounting for 70-80% of all breast cancer types (Haque et al., 2012). Triple negative breast cancer (TNBC) is a basal-like, aggressive subtype of breast cancer that accounts for 15-20% of other breast carcinomas, specifically among African-American young women (Sturtz et al., 2014). TNBC is identified by the absence of estrogen receptor, progesterone receptor, and overexpression of human epidermal growth factor receptor-2 (HR^-^/HER2^-^). Comparative to Luminal A and Luminal B, TNBC is highly recurrent and distantly metastatic breast cancer and irresponsive to commonly used hormone therapies to treat breast cancer due to lack of specific target sites (Qian et al., 2013).

Currently, chemotherapy, radiotherapy and hormone based remedies along with surgery (as first line therapy) has improved the five year survival rate of breast cancer patients (Ramezanpour et al., 2011). Besides this, the efficacy of presently available therapeutic drugs has been limited by notable side effects. Moreover, the heterogeneity of breast carcinoma imparts major challenges in the treatment and management of breast cancer. Tumor heterogeneity is one of the most important factors in the emergence of drug resistance in breast cancer (Ellsworth et al., 2017). In a large proportion of patients, drug resistance leads to tumor recurrence and metastasis. In addition, TNBC is highly associated with high metastasis to brain and lung in women under 50 years of age. Endocrine therapies are not successful in curing TNBC which has become an utmost clinical challenge. In the current scenario, novel therapeutic agents are needed to be identified for both HR^+^ and HR^-^ breast carcinomas that could evade drug resistance emergence and cancer heterogeneity with low cytotoxicity.

For breast cancer management, the concept of complementary and alternative medicinal (CAM) therapy along with conventional therapy is emerging that clearly advocates for the significance of plant based traditional drugs. Active compounds in herbal medicines may overcome drug resistance by targeting multiple signaling pathways. Cardiac glycosides (CGs) are diverse, natural compounds of steroidal framework with sugar portion at position 3 (C-3) and unsaturated lactone ring at position 17 (C-17). Cardenolides and bufadienolides are sub-groups of CGs containing five-membered unsaturated butyrolactone ring and six-membered unsaturated pyrone ring respectively (Maryam et al., 2018). CGs, extracts of *Digitalis purpurea L.*, are used to treat cardiac disorders like cardiac arrest, cardiac arrhythmias and heart congestions. Physiologically, these glycosides inhibit Na^+^/K^+^-ATPase pump which leads to boost sodium and calcium ions level inside the cell. In some recent findings, it has been suggested that several important cellular processes are regulated by additional modes of actions of Na^+^/K^+^-ATPase which highlights the novel potential role of cardiac glycosides in various diseases including cancer (Prassas and Diamandis, 2008).

Multiple factors like cell metabolism, survival, host defense, growth and differentiation are mediated by an important transcription factor identified as signal transducer and activator of transcription 3 (STAT3). It is activated indefatigably in cancer cells as compared to normal cells, provoking new approaches to inhibit STAT3 activation for the suppression of tumor growth. It has been disclosed that some of the current therapeutic drugs activate STAT3 can lead to resistance. Therefore, new drug discoveries in reverence to STAT3 inhibition would improve consequences of breast cancer treatment qualitatively, and more importantly, for both ER^+^ and ER^-^ breast cancer. In the present study, we have revealed the anticancer activity of cardiac glycoside “PSD-A” in MCF-7 and MDA-MB-231 breast cancer cell lines. Cytotoxic effect of these Na^+^/K^+^ inhibitors has been well known for many years but the molecular mechanism is not well understood. This study reflects that PSD-A induces apoptosis and autophagy and inhibits STAT3 activation in breast cancer cell lines. Moreover, we have cross checked the impact of STAT3 inhibition and cross talk between mitogen-activated protein kinase (MAPK) family proteins on apoptosis and autophagy. In some cases, autophagy maintains cancer cell survival and mediates resistance to therapies during metabolic stress conditions (Degenhardt et al., 2006). Therefore, we have disclosed the impact of autophagy on apoptotic activity of PSD-A.

## Materials and Methods

### Chemicals and Reagents

**Figure 1:**
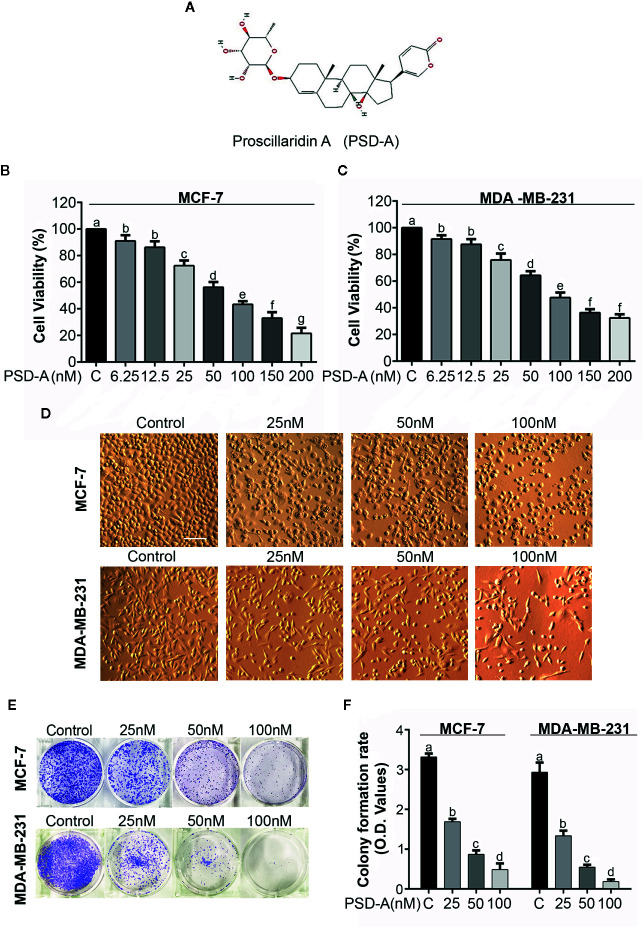
Antiproliferative and cytotoxic effect of PSD-A in breast cancer cells**. (A)** Chemical structure of PSD-A (Source: PubChem CID:222154) **(B, C)** MCF-7 and MDA-MB-231 cells were treated with indicated concentrations of PSD-A for 24 h. CCK-8 cell counting kit was used to measure cell viability. **(D)** Changes in morphology of MCF-7 and MDA-MB-231 cells were observed after 24 h PSD-A treatment using phase contrast microscope (Leica, DMIL LED). **(E)** MCF-7 and MDA-MB-231 cells were treated with indicated concentrations of PSD-A for 24 h and 300 cells/well were seeded into a 6-well plate within DMEM. After 10 days, formed colonies were fixed with 4% paraformaldehyde (PFA) and stained with crystal violet (CV). **(F)** Stain taken by the colonies was dissolved in methanol and optical density (OD) was measured at 595 nm. **(D)** Scale bar is 100 μm. **(B, C, F)** Data are expressed as Mean ± SD while all experiments were performed in triplicate independently. Columns with different superscript letters differ significantly (p < 0.05).

PSD-A (**Figure 1A**) with the highest purity grade of >98% was purchased from EXTRASYNTHESE (Genay, France). Dulbecco’s Modified Eagle’s Medium (DMEM) and fetal bovine serum (FBS) was purchased from Gibco (Eggenstein, Germany) while trypsin with or without EDTA was obtained from Gibco (Canada). Penicillin and streptomycin as well as acridine orange stain were purchased from Solarbio Co. Ltd. (Beijing, China). Cell counting kit-8 (CCK-8) was purchased from Bimake (China). Dimethyl sulfoxide (DMSO), phenyl methyl sulfonyl fluoride (PMSF) and protease inhibitor cocktail were obtained from Sigma-Aldrich (St. Louis, MO). Hoechst-33258 stain was purchased from Beyotime Institute of Biotechnology (Nanjing, China). Reactive oxygen species (ROS) assay kit, mitochondrial membrane potential assay kit (JC-1), annexin V-FITC apoptosis detection kit, crystal violet (CV) stain, fluo-3 AM and N-Acetyl-L-cysteine (NAC) were purchased from Beyotime Biotechnology (Haimen, Jiangsu, China). BAPTA-AM, SB203580 and SP600125 were purchased from Selleckchem (Munich, Germany) while epidermal growth factor (EGF) was procured from PeproTech (Rocky Hill, USA). Recombinant human STAT3 protein was procured from abcam (Cambridge, MA).

Primary antibodies for PARP, cleaved PARP, caspase-3, cleaved caspase-3, caspase-9, cleaved caspase-9, Bax, STAT3 and p-STAT3 (Tyr 705), proto-oncogene tyrosine-protein kinase SRC and p-SRC, Atg3, LC3 I/II, BiP/GRP78, AKT and p-AKT, p38 MAPK and p-p38 MAPK, c-Jun N-terminal kinase (JNK) and p-JNK, extracellular signal-regulated kinase (ERK1/2) and p-ERK1/2 were purchased from Cell Signaling Technology (Beverly, MA). Bcl-2, SHP-2, Beclin-1, ATF4, mTOR, p-mTOR and GAPDH were procured from Proteintech (Wuhan, China). Goat anti-rabbit and goat anti-mouse secondary antibodies were obtained from Sigma-Aldrich (St. Louis, MO).

### Cell Culture

Human MCF-7 and MDA-MB-231 breast cancer cell lines were purchased from American Type Culture Collection (Manassas, VA, USA). Cells were cultured in high glucose DMEM supplemented with 10% FBS, 100 units/mL penicillin and 100 μg/mL streptomycin. The cells were kept in a completely humidified atmosphere with 5% CO_2_ at 37°C.

### Cell Treatment and Cell Viability Assay

PSD-A was dissolved in DMSO with its final concentration of 0.5% and DMSO treated cells with the same concentration were used as control. A CCK-8 kit was used to determine cell viability according to the manufacturer’s instructions. Briefly, MCF-7 and MDA-MB-231 were seeded in 96-well microtiter plates with density of 3 ×10^3^ cells/well/100 µL and treated with different concentrations of PSD-A for 24h as indicated. Following treatments, 10 µL of CCK-8 solution was added to each well and kept for 3h at 37°C and OD was recorded at a wavelength of 450 nm by using a fluorescence microplate reader (Synergy neo HTS multimode microplate reader, BioTek). All experiments were performed in triplicate and cell viability was calculated using following formula.

Cell viability (%)=(A450 sample−A450 blank)/(A450 control−A450 blank)×100.

where “sample” shows optical density (OD) of drug treated cells with CCK-8 solution while “blank” represents OD value of culture medium with CCK-8 but without cells.

### Microscopic Observations of Cell Morphology

MCF-7 and MDA-MB-231 cells were cultured in 96-well plates for 24 h at 37°C and treated with indicated concentrations of PSD-A for next 24 h. Morphological changes in both cell lines were observed and then photographed by phase contrast microscope (Leica, DMIL LED).

### Clonogenic Assay

MCF-7 and MDA-MB-231 cells were treated with indicated concentrations of PSD-A for 24 h. Cells were washed, trypsinized and 500 cells/well were seeded into a 6-well plate in DMEM. Cells were allowed to form colonies at 37°C for 7 days in a humidified atmosphere. Old media was replaced with fresh DMEM after each 24 h. Once colonies were formed, cells were washed and fixed with 4% paraformaldehyde (PFA) for 15 min and stained with crystal violet for 20 min in the dark. Colonies were washed with PBS and photographed. Proliferative ratio of cells was enumerated by adding methanol in each well to dissolve crystal violet taken by colonies. Absorbance was measured at 595 nm by fluorescent spectrophotometer (Synergy neo HTS multi-mode microplate reader, BioTek).

### Hoechst 33258 Staining for Nuclear Morphology

MCF-7 and MDA-MB-231 cells were treated with indicated concentrations of PSD-A for 24 h. Cells were washed, collected and fixed in 4% PFA at room temperature for 30 min. Cells were washed twice with PBS and incubated with Hoechst stain 33258 (20 µg/mL) for 20 min in the dark at room temperature. Cells were collected, washed with PBS to remove extra stain and resuspended in PBS. Nuclear morphology of the cells was observed under a fluorescence microscope (Leica, DMI 4000B). Stained cells/100 cells were counted in three different fields to determine apoptotic percentage.

### Apoptosis Assay

Apoptotic effect of PSD-A was determined by annexin V-FITC/PI double staining apoptosis detection kit according to the manufacturer instructions. Briefly, MCF-7 and MDA-MB-231 cells were cultured in 6-well plates and treated with indicated concentrations of PSD-A for 24 h. Cells were washed with PBS, collected and resuspended in 1X binding buffer. The samples were incubated with 5 µL annexin V-FITC and 10 µL PI in the dark for 15 min and percentage of apoptosis was analyzed by flow cytometry (BD Accuri C^6^).

### Evaluation of Intracellular Reactive Oxygen Species (ROS) Generation

ROS generation was determined according to the manufacturer’s instruction by using ROS assay kit (Beyotime, China). Briefly, MCF-7 and MDA-MB-231 cells were seeded in 96-well plates and treated with indicated concentrations of PSD-A for 24 h and 50 nM for the indicated time constraints in presence or absence of NAC to evaluate ROS generation in dose and time dependent fashion respectively. Cells were then incubated with 2´, 7´-dichlorofluorescein–diacetate (DCFH-DA) dissolved in serum-free DMEM (1:1000) in the dark for 30 min. Cells were washed with DMEM and DCF fluorescence was measured by fluorescence microplate reader (Synergy neo HTS multi-mode microplate reader, BioTek) with excitation and emission wavelengths of 488 nm and 525 nm respectively.

### Determination of Mitochondrial Membrane Potential (ΔΨm)

Mitochondrial membrane potential (MMP) was determined by using mitochondrial membrane potential assay kit with JC-1 (Beyotime, China). Briefly, MCF-7 and MDA-MB-231 cells were treated with 25 nM, 50 nM and 100 nM concentrations of PSD-A for 24 h. Cells were washed with serum-free DMEM and incubated in JC-I working solution for 20 min in the dark at 37°C. After washing, cells were re-suspended with JC-1 dying buffer and JC-1 monomer fluorescence distribution (with excitation λ 490 nm and emission λ 530 nm) and j-aggregates (with excitation λ 525 nm and emission λ 590 nm) was measured using fluorescent spectrophotometer (Synergy neo HTS multimode microplate reader, BioTek). MMP of MCF-7 and MDA-MB-231 cells for control and treated groups was calculated by decrease in red/green fluorescence intensity ratio.

### Measurement of Intracellular Free Ca^+2^

Fluo-3 AM, a fluorescent probe, was used to detect intracellular free Ca^+2^ as described previously (Maryam et al., 2018). Briefly, cells were cultured in 96-well plates and treated with indicated concentrations of PSD-A in the presence or absence of BAPTA-AM for 24 h. Cells were washed and resuspended in serum-free medium. The samples were incubated with 5 µM Fluo-3 AM for 30 min in the dark. Cells were washed with PBS and analyzed at 480 nm excitation and 525 nm emission wavelengths using a microplate reader (Synergy neo HTS multimode microplate reader, BioTek).

### Surface Plasmon Resonance (SPR) Analysis

SPR analysis was performed as we mentioned earlier (Khan et al., 2020). Briefly, to validate direct interaction of PSD-A with STAT3, a series of PSD-A ascending concentration gradient from 39.0625 nM to 5000 nM was monitored on a C5 sensor chip immobilized with STAT3. PSD-A was injected with flow rate of 30 µL/min. Association and dissociation time was set to 90.1 sec and 90.4 sec respectively.

### Acridine Orange Staining

Acridine orange staining was performed as described previously (Kim et al., 2017). Briefly, cells were treated with different concentrations of PSD-A for 24 h. Cells were incubated with 1 µM acridine orange stain for 25 min in dark at 37°C. Cells were washed with PBS and images were taken using a fluorescence microscope (λ_ex_ = 488 nm, λ_em_ = 515 nm).

### Immunoblotting

MCF-7 and MDA-MB-231 cells were treated with indicated concentrations of PSD-A for indicated time in the presence or absence of NAC, BAPTA-AM, EGF, SP600125 and SB203580. Cells were collected and washed twice with cold PBS. After washing, cells were lysed with radio immunoprecipitation assay RIPA (Beyotime Biotechnology) supplemented with 2% sodium fluoride (NaF) and 1% phenylmethylsulfonyl fluoride (PMSF) on ice for 30 min. Cells were centrifuged at 12000 rpm for 15 min and supernatant was collected in ice chilled tubes. A BCA protein assay kit (Beyotime Biotechnology) was used to determine protein concentrations by spectrophotometer (Synergy neo HTS multimode microplate reader, BioTek).

Equal amounts of proteins (30-40 µg) were loaded and separated on 10-12% sodium dodecyl sulfate polyacrylamide gel electrophoresis (SDS-PAGE). Proteins were then transferred to polyvinylidene difluoride (PVDF) membranes. After transfer, PVDF membranes were blocked in 5% skim milk for 1 h. After three times wash with Tris-buffered saline-tween (TBST), membranes were incubated in corresponding primary antibodies at 4°C overnight. Membranes were washed with TBST three times and incubated with HRP-conjugated goat anti-rabbit IgG or goat anti-mouse IgG secondary antibodies for 1 h at room temperature. After washing membranes with TBST, ECL plus chemiluminescence kit was used to detect immune-reactive bands in DNR bioimaging system MicroChemi 4.2. All immunoblots were repeated three times and GAPDH was measured as loading control in each blot. Variations in proteins expression was determined using ImageJ software.

### Statistical Analysis

Results were expressed as mean ± SD for three different experiments and compared statistically with untreated (control) or within treated groups using one-way ANOVA followed by Tukey’s Multiple Comparison Test. For comparison of only two groups, student *t*-test was used. *P* < 0.05 was measured to be statistically significant.

## Results

### PSD-A Induces Anti-Proliferative and Cytotoxic Effect in Breast Cancer Cells

MCF-7 (triple positive) and MDA-MB-231 (triple negative) breast cancer cells were used in particular to evaluate the anti-proliferative and cytotoxic effects of PSD-A. A CCK-8 cell counting kit was used to measure cell viability of both MCF-7 and MDA-MB-231 cell lines in the presence or absence of PSD-A. We found a remarkable dose-dependent decrease in cell viability percentage among PSD-A treated groups compared to the untreated (**Figures 1B, C**). IC_50_ values for MCF-7 and MDA-MB-231 cells at the 24 h time point were found to be approximately 40 nM and 38 nM respectively, evaluating PSD-A to be equally effective for both triple positive and triple negative breast cancer cell lines. Therefore, we preferred both MCF-7 and MDA-MB-231 cells for further comparative mechanistic study. 25, 50 and 100 nM were the most suitable PSD-A concentrations for both cells among whole concentration gradient from 6.25 to 200 nM.

To explore the effect of PSD-A on morphology of breast cancer cells, we exposed both cell lines to the indicated concentrations of the drug for 24 h. We observed that PSD-A induced several morphological changes typically related to the cell death, i.e. lost cellular geometry, rounded in shape and floating on the media surface (**Figure 1D**). Further, we performed clonogenic assay to evaluate growth inhibitory and anti-proliferative effect of PSD-A in MCF-7 and MDA-MB-231 cells. For the purpose, we exposed cells to the indicated concentrations of PSD-A and allowed the treated cells for several days to make colonies. Compared to the normal, we found a significant decrease in the number of colonies (**Figure 1E**). We further quantified the rate of cell proliferation by dissolving crystal violet stain (attained by the cells) in methanol. As shown in **Figure 1F**, a significant decrease was found in the uptake of crystal violet (CV) stain in treated cells compared to the untreated. Collective data of CCK-8 assay, morphological examination and clonogenic assay reveal that PSD-A inhibits proliferation and induces cytotoxic effect in MCF-7 and MDA-MB-231 breast cancer cell lines.

### PSD-A Induces Mitochondrial Apoptotic Cell Death *via* ROS Generation and Intracellular Ca^+2^ Accumulation in MCF-7 and MDA-MB-231 Breast Cancer Cells

**Figure 2:**
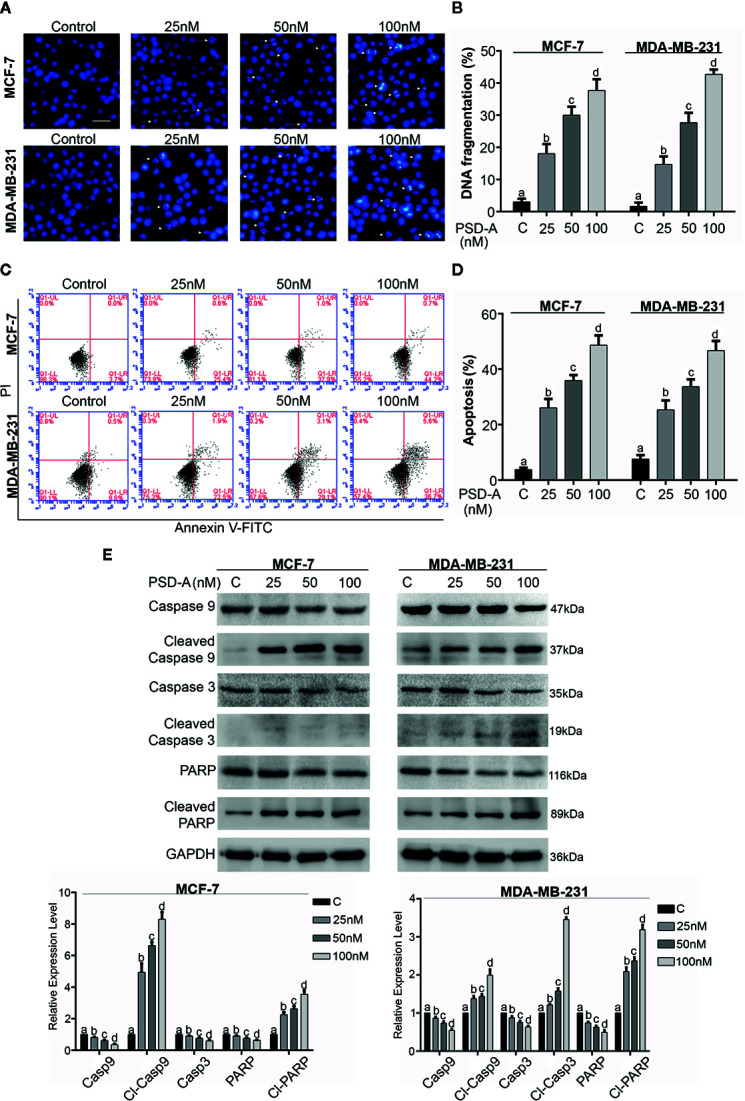
Induction of apoptosis in breast cancer cells. **(A)** MCF-7 and MDA-MB-231 cells were treated with indicated concentrations of PSD-A for 24 h and stained with Hoechst 33258 for nuclear morphological changes. Scale bar is 100 μm. **(B)** Among 100 cells in three different fields, cells with stained nuclei were counted to determine percentage of DNA fragmentation. **(C, D)** Breast cancer cells were treated with 25, 50 and 100 nM concentrations of PSD-A for 24 h, stained with Annexin V-FITC and PI and analyzed by flow cytometry. Percentage of apoptosis was presented in graphical format. **(E)** After PSD-A treatment for 24 h, protein extracts were prepared and analyzed by western blotting to measure expression level of caspase- 9, cleaved caspase-9, caspase-3, cleaved caspase-3, PARP and cleaved PARP; the hallmarks of apoptosis. GAPDH was used as loading control. **(B, D, E)** Data are expressed as Mean ± SD while all experiments were performed in triplicate independently. Columns with different superscript letters differ significantly (p < 0.05).

PSD-A is well-known to induce apoptotic cell death in various cancer types (He et al., 2018; Maryam et al., 2018). More specifically, CGs are exposed to be involved in induction of apoptosis *via* DNA fragmentation (McConkey et al., 2000). In order to ascertain mode of cell death, we performed Hoechst-33258 staining to analyze DNA fragmentation in PSD-A treated breast cancer cells compared to the non-treated. We found intensified DNA fragmentation in PSD-A treated cells in a dose-dependent manner as shown in **Figures 2A, B**. PSD-A induced apoptotic cell death was further confirmed by flow cytometry. Both cell lines, MCF-7 and MDA-MB-231, were treated with the indicated concentration of PSD-A for 24 h and stained with annexin V-FITC and PI for detection of apoptosis. Flow cytometry analysis revealed the substantial increase in percentage of annexin V-positive cells (early apoptosis) in both MCF-7 and MDA-MB-231 cells in dose-dependent fashion (**Figures 2C, D**). Further, we proved PSD-A induced apoptotic cell death in breast cancer cells *via* analyzing expression of apoptotic hallmarks i.e. cleavage of caspase-9, caspase-3 and poly (ADP ribose) polymerase (PARP) along with total caspase-9, caspase-3 and PARP. PSD-A increased the expression of cleaved caspase-9, cleaved caspase-3 and cleaved PARP in MDA-MB-231 cells. Similarly, cleaved caspase-9 and cleaved PARP was significantly increased in PSD-A treated MCF-7 cells, however, caspase-3 was not detected during western blotting analysis as MCF-7 human breast cancer cells are deficient of caspase-3 (**Figure 2E**). Total caspase-9, caspase3, and PARP were decreased in dose-dependent fashion. Above collected data indicated that PSD-A induces apoptosis in breast cancer cells in dose-dependent manner.

**Figure 3:**
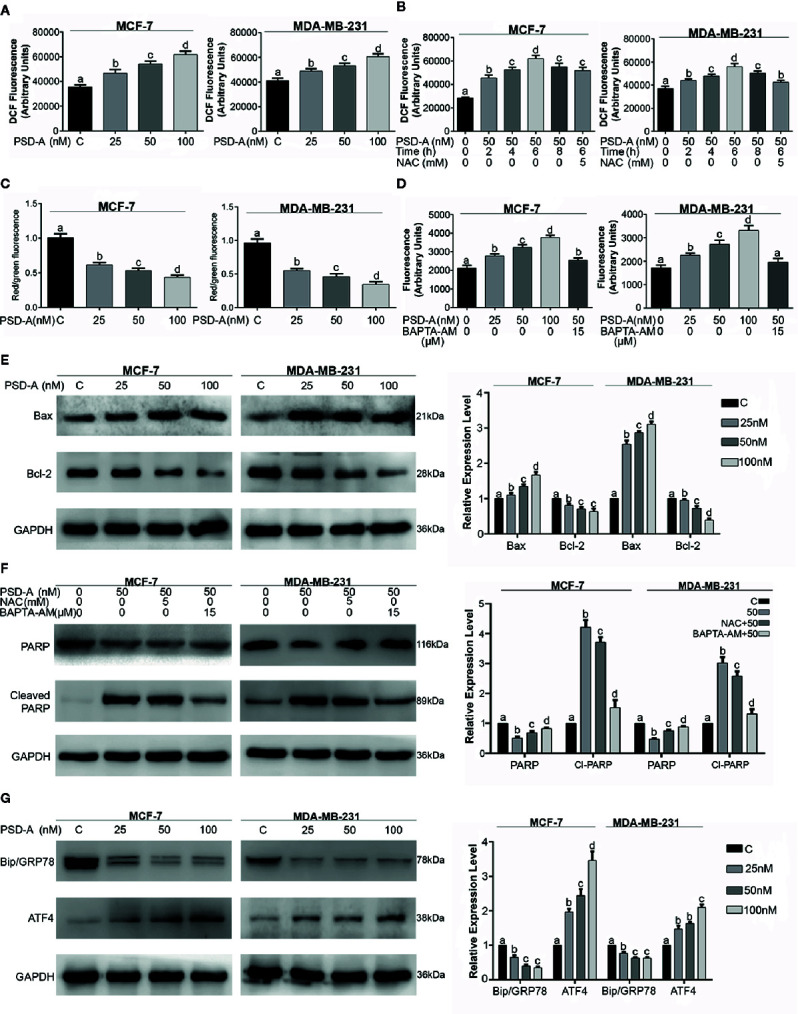
PSD-A induces mitochondrial apoptosis dependent of ROS and intracellular Ca^+2^ accumulation. **(A)** MCF-7 and MDA-MB-231 cells were treated with indicated concentrations of PSD-A for 24 h and stained with DCFH-DA to determine dose-dependent intracellular ROS level. **(B)** Cells were treated with PSD-A (50 nM) for 2, 4, 6 (in presence and/or absence of NAC) and 8 h. Cells were stained with DCFH-DA and ROS generation was measured compared to the control. **(C)** Cells were treated with 25, 50 and 100 nM of PSD-A for 24 h and stained with JC-1 solution to measure MMP level. **(D)** MCF-7 and MDA-MB-231 cells were treated with PSD-A for 24 h and intracellular free Ca^+2^ was measured using Fluo-3 AM. **(E)** Cells were exposed to the indicated concentrations of PSD-A for 24 h and pro-apoptotic Bax and anti-apoptotic Bcl-2 protein expressions were determined by western blotting. **(F)** Cells were treated with PSD-A (50 nM) in presence or absence of NAC (5 mM) and BAPTA-AM (15 µM), PARP and cleaved PARP expression was measured by western blotting. **(G)** Cells were treated with PSD-A for 24 h and expression of GRP78 and ATF4 was determined by western blotting. **(E–G)** GAPDH was used as internal control. **(A–G)** Graphical data are expressed as Mean ± SD while all experiments were performed in triplicate independently. Columns with different superscript letters differ significantly (p < 0.05).

Next, we were interested to know whether PSD-A induces ROS generation in MCF-7 and MDA-MB-231 cells in both dose and time-dependent manner. For the purpose, we treated cells with indicated concentration of PSD-A for indicated time intervals and stained the cells with 2´, 7´-dichlorofluorescein-diacetate (DCFH-DA). We found significant and gradual increase in ROS level in both cell types treated with ascending dose gradients of PSD-A (**Figure 3A**). Likewise, we observed significant elevation of ROS in time-dependent fashion with early rise at 2 h treatment, reaching maximum at 6 h and starting to decrease at 8 h treatment. However, pretreatment with NAC (5mM), a ROS scavenger, partially inhibited PSD-A induced ROS generation at the highest time point of 6 h as shown in **Figure 3B**. Following measurement of ROS levels, we determined change in mitochondrial membrane potential (ΔΨm) to authenticate effect of PSD-A in mitochondrial apoptosis. As indicated in **Figure 3C**, we noticed significant decrease in MMP in both MCF-7 and MDA-MB-231 cells. In previous studies, it has been shown that PSD-A increases intracellular Ca^+2^ level (Li et al., 2018). Therefore, we determined the effect of PSD-A in MCF-7 and MDA-MB-231 cells by using Fluo-3AM. Our data demonstrated that PSD-A increased intracellular Ca^+2^ level in both cell types in a dose-dependent manner. However, pretreatment of BAPTA-AM (15 µM), a Ca^+2^ chelator, significantly inhibited intracellular Ca^+2^ accumulation in the presence of indicated concentration of PSD-A (**Figure 3D**).

Henceforth, augmented ROS and dissipated MMP levels imposed us to find the effect of PSD-A over Bcl-2 family proteins modulation. Therefore, we measured the expression of Bax and Bcl-2 proteins in PSD-A treated MCF-7 and MDA-MB-231 cells compared to the untreated. We found a significant increase in the expression of Bax while also finding a substantial decrease in Bcl-2 proteins in both cell types (**Figure 3E**). Above mentioned data suggests that PSD-A induces mitochondrial apoptosis in breast cancer cells. After finding parallel targeted hallmarks for mitochondrial apoptosis, we were interested to know whether ROS generation and intracellular Ca^+2^ oscillation takes part in PSD-A-induced apoptosis or not. Hence, we measured the expression of PARP and cleaved PARP in the presence or absence of NAC and BAPTA-AM. We found that pretreatment of NAC decreased PSD-A induced PARP cleavage up to a low significant level while that of BAPTA-AM significantly decreased PARP cleavage in both cell types indicating the dynamic role of intracellular Ca^+2^ in apoptosis (**Figure 3F**).

### PSD-A Promotes ER Stress in MCF-7 and MDA-MB-231 Breast Cancer Cells

Immunoglobulin heavy chain binding protein (BIP), also known as GRP78, is the main regulator of ER function that facilitates and assembles the folding of new proteins, targets misfolded proteins for degradation and controls the activation of transmembrane ER stress sensors (Yang et al., 2016). In case of ER stress, GRP78 expression is elevated, correlates with tumor development and counteracts the apoptosis-inducing potential of ER stress (Mhaidat et al., 2009). Due to intracellular Ca^+2^ imbalance and its role in apoptosis, we hypothesized the possible role of PSD-A in ER stress chaperones modulation. Surprisingly, we found a substantial inhibitory effect of PSD-A on cytoprotective ER chaperone GRP78 in both cell lines in dose-dependent fashion. Moreover, we noticed significant increase in expression of activating transcription factor 4 (ATF4) that assumes a positive role of PSD-A in the initiation of ER stress (**Figure 3G**).

### PSD-A Inhibits Constitutive and EGF-Induced STAT3 Activation

**Figure 4:**
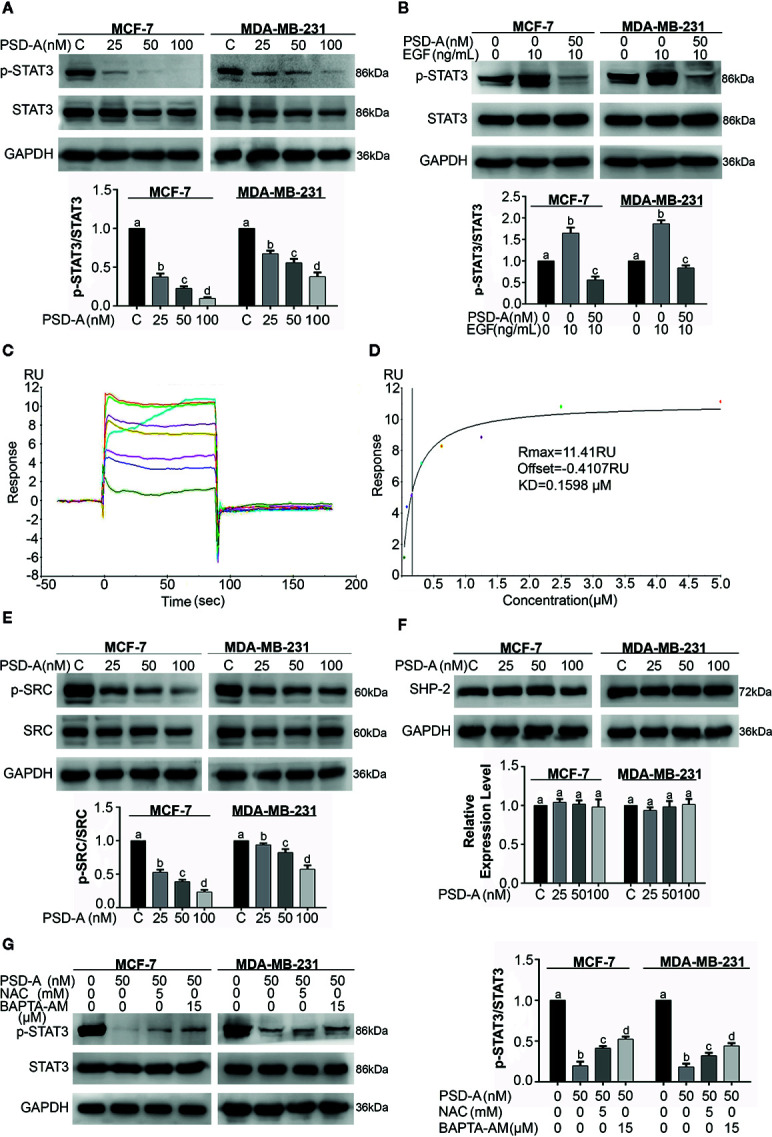
PSD-A inhibits constitutive and EGF-induced STAT3 activation *via* ROS generation and intracellular Ca^+2^ accumulation. **(A)** Cells were treated with indicated concentrations of PSD-A for 24 h and protein extracts were prepared using RIPA buffer. Expression of p-STAT3/STAT3 was measured by western blotting. **(B)** The cells were pretreated with the PSD-A (50 nM) for 4 h and stimulated with EGF (10 ng/mL) for 10 min. Whole cell lysates were subjected to western blotting to determine the p-STAT3/STAT3 expression. **(C, D)** Time and dose-dependent binding affinity of PSD-A with STAT3 was evaluated by SPR analysis **(E)** Expression of p-SRC/SRC and **(F)** SHP-2 were measured by western blotting. **(G)** Cells were exposed to PSD-A (50 nM) in presence or absence of NAC (5 mM) and BAPTA-AM (15 µM) for 24 h and expression of p-STAT3/STAT3 was measured by western blotting. **(A, B, E–G)** GAPDH was used as internal control. Graphical data are expressed as Mean ± SD while all experiments were performed in triplicate independently. Columns with different superscript letters differ significantly (p < 0.05).

Janus Kinase (JAK)-Signal transducer and activator of transcription 3 (STAT3) pathway is vigorously triggered in multiple cancer types leading to cell survival, proliferation and resistance to the anticancer therapeutics (Shou et al., 2016). Thus, we investigated the role of PSD-A in STAT3 inhibition in MCF-7 and MDA-MB-231 cell lines. As shown in **Figure 4A**, PSD-A significantly inhibited STAT3 activation by suppressing its phosphorylation at tyrosine 705 in both triple positive and triple negative breast cancer cells. Next, we used epidermal growth factor (EGF) to induce STAT3 activation in MCF-7 and MDA-MB-231 cells. As shown in **Figure 4B**, EGF (10 ng/mL) treatment resulted in STAT3 activation. However, pretreatment with PSD-A significantly inhibited EGF-induced STAT3 activation. Our data indicate that PSD-A inhibits both constitutive and EGF-induced STAT3 activation. In a previous study, we have simulated by molecular docking that PSD-A could directly bind STAT3 (Maryam et al., 2018). Here, we used the SPR technique to verify whether PSD-A directly targets STAT3 in preventing EGF-induced STAT3 activation, as given in **Figures 4C, D**. We observed a time- and dose-dependent binding of PSD-A with STAT3 with an equilibrium dissociation constant (KD) of around 0.1598 µM. Our results provide enough evidence for validation of direct interaction of PSD-A with STAT3.

As SRC family kinases and MAPKs are the upstream signaling molecules for activation of STAT3, we therefore measured expression levels of p-SRC and SRC. We found a significant decrease in expression levels of p-SRC in PSD-A treated cells compared to the untreated (**Figure 4E**). Similarly, we measured the expression level of one of the members of protein tyrosine phosphatases (PTPs); SHP-2 that negatively regulates STAT3 activation. We did not find any significant change in SHP-2 expression as indicated in **Figure 4F**. The data showed that PSD-A mediated SRC inhibition might be associated with inhibition of STAT3 activation.

Previous studies clearly indicate that ROS generation and intracellular Ca^+2^ oscillation is involved in STAT3 inhibition (Zhang et al., 2015; Lu et al., 2017). Therefore, we were interested to know their impact in PSD-A induced STAT3 inhibition in MCF-7 and MDA-MB-231 cells. As shown in **Figure 4G**, pretreatment of NAC and BAPTA-AM invigorated STAT3 activation in PSD-A treated cells up to some extent. This data suggests that inhibition of STAT3 activation by PSD-A is slightly dependent on ROS generation as well as Ca^+2^ oscillation inside the cells.

### PSD-A Induces Autophagy in MCF-7 and MDA-MB-231 Breast Cancer Cells

**Figure 5:**
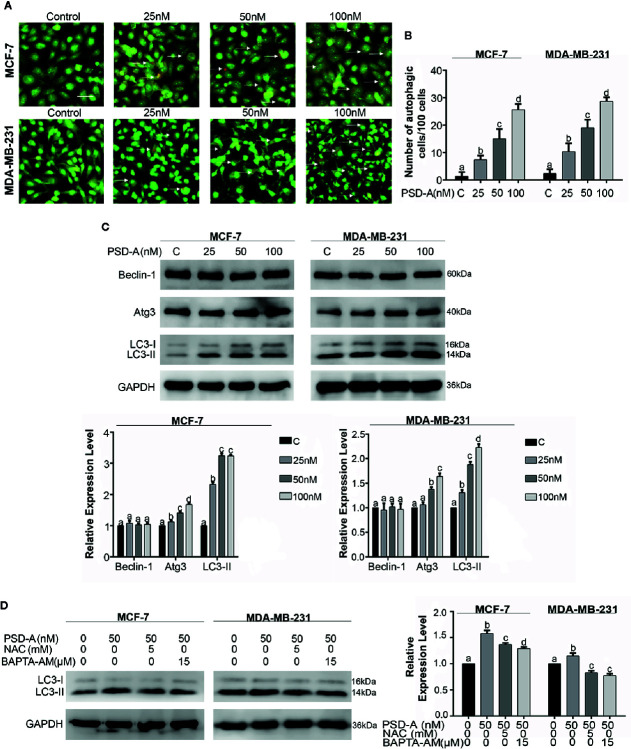
PSD-A induces autophagy in MCF-7 and MDA-MB-231 cells. **(A)** Cells were exposed to PSD-A for 24 h, stained with acridine orange (AO) stain and photographed under fluorescence microscope. Scale bar is 100 μm. **(B)** Three different fields were selected and number of AO stained cells were counted/100 cells. **(C)** After exposing cells to indicated concentrations of PSD-A for 24 h, protein lysates were prepared and protein expression of Beclin-1, Atg3, and LC3-I/LC3-II was determined by western blotting. **(D)** Cells were exposed to PSD-A (50 nM) in presence or absence of NAC (5 mM) and BAPTA-AM (15 µM) for 12 h and expression of LC3-I/LC3-II was measured by western blotting. **(C, D)** GAPDH was used as loading control. **(B–D)** Graphical data are expressed as Mean ± SD while all experiments were performed in triplicate independently. Columns with different superscript letters differ significantly (p < 0.05).

In this study, we explored the competence of PSD-A to induce autophagy for the first time in any cancer type. Foremost, we treated MCF-7 and MDA-MB-231 cells with indicated concentrations of PSD-A for 24 h and stained with acridine orange dye. To measure intense autophagic induction, we counted acridine orange stained cells per 100 cells in 3 different fields. Acridine orange is an organic basic dye that enters through plasma membrane of the cell and emits orange light upon protonation in acidic parts of the cells i.e. lysosomes. In our experiments, PSD-A treated cells indicated increased autophagic vacuolization compared to the untreated in a dose-dependent way as shown in **Figures 5A, B**. To further validate autophagic induction in breast cancer cells, we measured the protein levels of Beclin-1, Atg3, and the conversion of LC3-I to LC3-II which are the hallmarks of autophagy induction. Surprisingly, we did not find any obvious change in expression of Beclin-1; the key regulator of autophagy. However, the expression of Atg3 and conversion of LC3-I to LC3-II was significantly increased in PSD-A treated cells (**Figure 5C**).

Similar to the apoptosis, autophagy is triggered by ER stress and ER stress-dependent Ca^+2^ release (Høyer-Hansen and Jäättelä, 2007). After verifying immersion of ROS and Ca^+2^ oscillation in apoptosis, we were interested to find their role in autophagy. As shown in **Figure 5D**, pretreatment of NAC and BAPTA-AM reduced LC3 expression in MCF-7 and MDA-MB-231 cells providing enough evidence in support of their role in the initiation of autophagy. However, further detailed mechanistic study is needed to explore Ca^+2^ induced autophagy in PSD-A treated cells.

### MAPK Signaling Pathway Controls the Balance of PSD-A Induced Apoptosis and Autophagy in MCF-7 and MDA-MB-231 Cells

**Figure 6:**
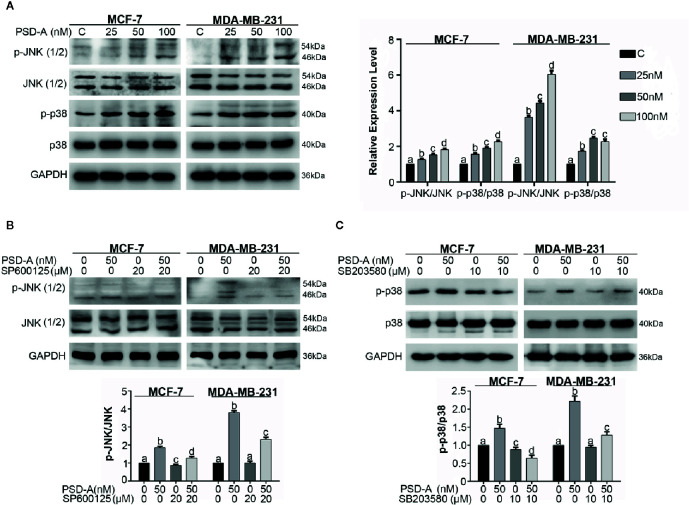
PSD-A induces JNK and p38 activation. **(A)** MCF-7 and MDA-MB-231 cells were treated with indicated concentrations of PSD-A for 8 h and expression level of p-JNK/JNK and p-p38/p38 was measured by western blotting. **(B)** Cells were treated with PSD-A (50 nM) for 8 h in presence and/or absence of SP600125 (20 µM) and protein expression for p-JNK/JNK was measured by western blotting. **(C)** Cells were treated with PSD-A (50 nM) for 8 h in presence and/or absence of SB203580 (10 µM) and protein expression for p-p38/p38 was measured by western blotting. **(A–C)** GAPDH was used as internal control. Graphical data are expressed as Mean ± SD while all experiments were performed in triplicate independently. Columns with different superscript letters differ significantly (p < 0.05).

The MAPK pathway is activated by extracellular stimuli i.e. UV radiations and ER stress and converts the stimulus to a wide range of cellular responses. More specifically, JNK plays a dual role in the cell’s fate, promoting cell survival on one hand and cell death on another (Tournier, 2013). Therefore, we were interested in elaborating on the effect of PSD-A treated cells on the MAPK signaling pathway. First, our data indicate that PSD-A significantly increased phosphorylation of JNK and p38 without affecting total JNK and p38 in both cell lines (**Figure 6A**). Secondly, pretreatment of commercial inhibitors SP600125 (JNK inhibitor) and SB203580 (p38 inhibitor) successfully inhibited their phosphorylation confirming a definite inhibitory effect as indicated in **Figures 6B, C**.

**Figure 7:**
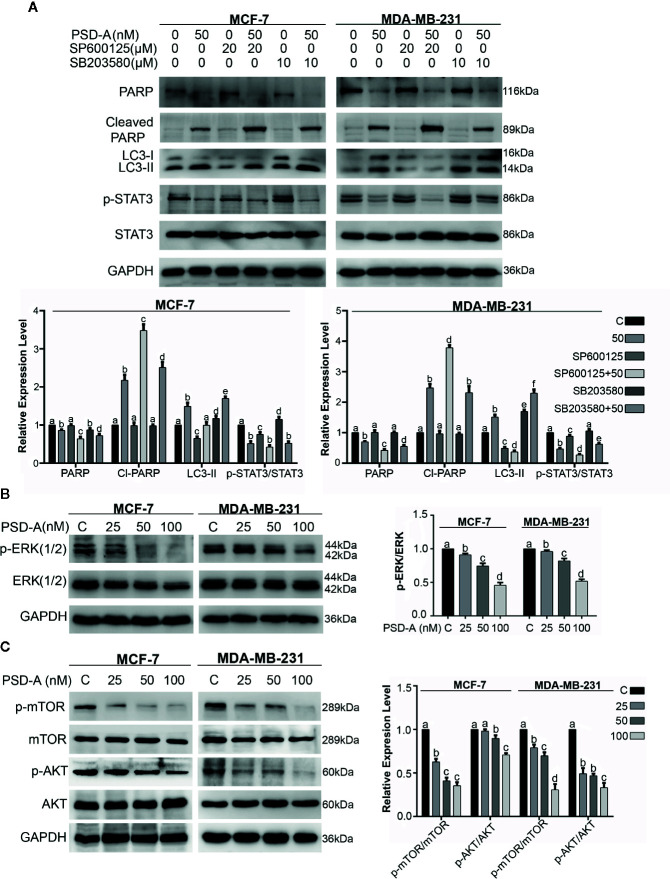
Effect of PSD-A over MAPK pathway and mTOR/AKT pathways in breast cancer cells. **(A)** Cells were treated with PSD-A (50 nM) in presence and/or absence of SP600125 (20 µM) and SB203580 (10 µM) for 8 h and expression level of PARP, cleaved PARP, LC3-I/LC3-II and p-STAT3/STAT3 was measured. **(B)** Cells were treated with PSD-A for 24 h and phosphorylation of ERK1/2 was determined by western blotting. **(C)** Cells were treated with PSD-A for 24 h and phosphorylation of mTOR and AKT was determined by western blotting. **(A–C)** GAPDH was used as internal control. Graphical data are expressed as Mean ± SD while all experiments were performed in triplicate independently. Columns with different superscript letters differ significantly (p < 0.05).

In recent studies, it has been suggested that MAPK, especially p38 MAPK and JNK, play a vital role in cross talk between apoptosis and autophagy (Sui et al., 2014; Moosavi et al., 2018). Besides, autophagy has a dual role; pro-death and pro-survival, depending on the cell type and stimuli (Kondo et al., 2005). For that reason, we had a concern to know the role of JNK and p38 in PSD-A induced apoptosis and autophagy. In our data, JNK inhibition by SP600125 promoted PSD-A initiated PARP cleavage leading to the apoptotic activity and reduced autophagic response in the cells by preventing LC3 expression. Furthermore, SP600125 enhanced STAT3 inhibition in PSD-A treated cells that signifies the role of STAT3 inhibition endorsing apoptosis. Also, to amplify role of p38 in PSD-A induced apoptosis and autophagy, we determined the levels of apoptosis and autophagic markers under pretreatment of SB203580. Surprisingly, 10 µM SB203580 pretreatment resulted in slight enhancement of PARP cleavage in MCF-7 cells only and conversion of LC3 in both cell types, nevertheless, pretreatment of SB203580 slightly restored PSD-A induced STAT3 inhibition in MDA-MB-231 cells (**Figure 7A**). All above outcomes designate that inhibition of JNK-induced autophagy promotes apoptosis in PSD-A treated breast cancer cells.

Further, to understand the underlying mechanism, we extended our study and measured the expression of p-ERK/ERK, p-mTOR/mTOR and p-AKT/AKT. As shown in **Figure 7B**, PSD-A decreased phosphorylation of ERK1/2 in a dose-dependent manner after 24 h treatment. AKT/mTOR signaling is one of the main regulatory pathway in cancer cells that negatively regulates autophagy (Liu et al., 2019). Our results showed that PSD-A decreased the expression of p-mTOR/mTOR and p-AKT/AKT in dose-dependent way after 24 h of drug exposure (**Figure 7C**). From all above data, we assume that PSD-A induces JNK and p38 MAPK activation where JNK leads to induce autophagy *via* inhibiting AKT/mTOR signaling pathway and p38 MAPK controls the cross talk of MAPKs in PSD-A induced apoptosis and autophagy.

## Discussion

Breast cancer, the most common type of cancer among women as well as second leading cause of cancer related mortality, has become a serious concern to global researchers (Karia et al., 2018). Surgery and radiotherapy are the first possible approaches to eradicate breast cancer but these tactics have severe side effects. Chemotherapy along with radiotherapy has already improved the 5-year survival rate in breast cancer patients (Nooshinfar et al., 2016) but hormone-independent breast cancer type is highly metastatic and more resistant to chemotherapy, limiting the effectiveness in therapy of these cancer types (Siegel et al., 2012). Therefore, it is important to identify new natural compounds with promising anticancer activity in ER-positive as well as ER-negative breast cancer types with endurable side effects.

CGs, primarily used in clinics for treatment of cardiac arrest, are now well-known for their antitumor activity by targeting multiple signaling pathways in various cancer types (Slingerland et al., 2013). PSD-A, bufadienolide cardiac glycoside, is famous for its anticancer property including breast cancer but the exact underlying mechanism still needs to be explored. More importantly, current ongoing cytoprotective responses of multiple cancer types to the CGs therapies *in vitro* have been noticed (Sun et al., 2018) that have forced us to elaborate with deep mechanistic study in both ER-positive and ER-negative breast cancer cells. In this study, we have investigated the anticancer mechanism of PSD-A in MCF-7 and MDA-MB-231 breast cancer cells. Some interesting underlying aspects were disclosed in this study that will not only augment PSD-A to be among the best possible therapeutics for curing breast cancer in the future but also will be imposed to enhance sensitivity for currently available drugs.

Firstly, PSD-A has been shown to exert cytocidal effect in MCF-7 and MDA-MB-231 cells in extremely low concentrations of 25, 50 and 100 nM by interacting with several novel cellular targets. Some of the unique characteristics of PSD-A disclosed in this study include, generation of ROS and intracellular Ca^+2^ accumulation leading to mitochondrial apoptosis, DNA fragmentation, mitochondrial membrane potential dissipation, caspases and PARP cleavages, Bax/Bcl-2 proteins modulation and generation of ER stress *via* inhibition of cytoprotective ER chaperone GRP78. In addition to all of the above, PSD-A was found to be involved in the inhibition of constitutive and EGF-induced STAT3 activation in breast cancer cells *via* targeting upstream tyrosine kinases. For the first time, PSD-A was unveiled to induce autophagy in any cancer type. In the current study, the role of MAPK signaling pathways in mediation of apoptosis and autophagy has also been documented.

Prior to the mentioned mechanistic study, we exposed MCF-7 and MDA-MB-231 cells to different concentrations of PSD-A and checked the cell viability. We observed a very significant cytotoxic effect of PSD-A on both cell types; thus, we sustained our study with both ER-positive and ER-negative representative cell lines. PSD-A has been well-known for its anti-proliferative and cytotoxic effect in different cancer types including lung cancer (Li et al., 2018; Maryam et al., 2018), prostate cancer (He et al., 2018) and more importantly in breast cancer along with the effect of various other CGs (Winnicka et al., 2008). To elucidate the precise cytotoxic effect of PSD-A on breast cancer, we observed obvious cytocidal morphological changes in treated cell lines. We performed a clonogenic assay to check the proliferative ability of cells under PSD-A treatment. We observed a significant decrease in the number of colonies in both cells representing anti-proliferative ability of PSD-A in breast cancer cells.

CGs inhibit Na^+^/K^+^ ATPase pump resulting in an increased level of Ca^+2^ inside the cell. This increase in Ca^+2^ level is the main driving force to rehabilitate cardiac contractile ability of heart muscles to prevent cardiac arrest (Calderón-Montaño et al., 2014). More recently, it has been justified that intracellular Ca^+2^ elevation and high ROS level induces apoptosis in cancer cells while intracellular Ca^+2^ intensification can also induce autophagy (Smaili et al., 2012; Li et al., 2018; Moosavi et al., 2018). In our study, we used NAC (ROS scavenger) and BAPTA-AM (a calcium chelator) to illuminate the role Ca^+2^ and ROS in PSD-A induced apoptosis and autophagy. Ca^+2^ is a universal intracellular messenger which regulates functional viability of mitochondria, ER and lysosomes (Berridge et al., 2000). In mitochondria, an elevated Ca^+2^ level induces more ATP production *via* increasing electron transport chain (ETC) activity. This rise is accompanied by increased leakage of free electrons resulting in formation of free superoxide ions. These free radicles, peroxides and oxygen molecules are collectively known as reactive oxygen species (ROS). After accumulation of ROS, it oxidizes cellular components including nucleotides of DNA. Thus, mitochondrial Ca^+2^ plays an interesting role in the mediation of survival and death pathways. The high Ca^+2^ level dissipates the mitochondrial membrane potential and leads to initiate intrinsic apoptotic pathway (Chandra et al., 2006). In this study, we established that PSD-A elevated Ca^+2^ and ROS level and induced mitochondrial apoptosis in MCF-7 and MDA-MB-231 cells *via* intracellular Ca^+2^ load and ROS generation. We measured expression levels of intrinsic apoptotic markers i.e. cleaved caspase-9, cleaved caspase-3 and cleaved PARP. We found a significant increase in expression of these biomarkers. Moreover, we determined mitochondrial membrane potential and the expression of Bax and Bcl-2 proteins; imperative markers for mitochondrial apoptosis. Dissipative change in MMP along with Bax/Bcl-2 proteins modulation was observed in treated cells. We justified the role of Ca^+2^ and ROS in apoptosis by using NAC and BAPTA-AM which significantly restored PSD-A induced PARP cleavage.

We further assessed the role of PSD-A over ER chaperone. ER acts as a calcium reservoir and uses calcium for communication with other cell organelles. It is well-known that disturbance in ER calcium balance leads to extensive and irreversible damage and activates ER-specific cell death pathways (Smaili et al., 2012). GRP78, an ER chaperone, is involved in ER stress response and over expressed specifically in case of inhibition of ER ATPase and calcium depletion in ER (Garcia-Prieto et al., 2013). In contrast, PSD-A significantly inhibited GRP78 and increased ATF4 expression in dose- dependent fashion suggesting that PSD-A may not interact with ER ATPase. However, further in-depth study is required to understand the exact underlying mechanism of inhibition of GRP78 by PSD-A.

Until now, constitutive activation of STAT3 has been reported in numerous studies resulting into cancer proliferation, survival, progression and chemo-resistance (Kim and Yoon, 2016). Aberrant STAT3 activation is facilitated by irregularities in non-receptor tyrosine kinases i.e. JAKs and SRC as well as MAPKs and PTPs (Garcia et al., 2001; Chun et al., 2015). In previous studies, PSD-A has been found to inhibit STAT3 activation in prostate cancer (He et al., 2018) and A549 lung cancer cells (Maryam et al., 2018). In our data, PSD-A inhibited STAT3 activation by preventing phosphorylation of SRC. PSD-A did not affect PTPs like SHP-2. Impact of PSD-A over TPA and IL-6 induced STAT3 activation has been disclosed previously in lung cancer (Maryam et al., 2018). In this study, we determined that PSD-A is capable of inhibiting EGF-induced STAT3 activation in breast cancer cells. Moreover, Yan Lu and his coworkers have already reported that intracellular Ca^+2^ homeostasis restores STAT3 activation and protects BV2 microglia against hypoxia-induced inflammation and apoptosis (Lu et al., 2017). Likewise, the role of oxidative stress in the inhibition of STAT3 activation in lung cancer has already been conferred (Maryam et al., 2017). In this study, we determined for the first time that inhibition of STAT3 activation relies on Ca^+2^ disbalance and ROS generation in PSD-A treated breast cancer cells. For this purpose, we pretreated cells with BAPTA-AM and NAC respectively and then exposed to PSD-A for the indicated time period. We observed that calcium chelator and ROS scavenger reinstated the STAT3 activation up to a slight significant level suggesting role of Ca^+2^ and ROS in inhibition of STAT3 activation.

CGs are well-known for their ability to induce autophagy in various cancer types (Wang et al., 2012; Hu et al., 2014). In this study, PSD-A has been unveiled for the first time to induce autophagy in breast cancer cells. For the purpose, we determined the expression level of different autophagic markers, more specifically LC3 I/II. Formation of acidic vesicular organelles (AVOs) is one of the hallmarks in autophagy sequester through cytoplasmic proteins. We stained acidic vesicles inside the autophagic cells and determined the higher rate of autophagy in PSD-A treated cells. The evidences suggest that calcium- induced-ER stress is the suitable condition for initiation of autophagy (Shi et al., 2011). Moreover, ROS- dependent autophagy has previously been well-noticed in various cancers (Wu et al., 2018; Yang et al., 2018). Therefore, we were interested to explore whether PSD-A induced autophagy is ROS and Ca^+2^-dependent. By using NAC and BAPTA-AM, we were able to declare that PSD-A induces autophagy *via* ROS generation and Ca^+2^ buildup inside and cell.

The role of MAPK signaling pathways has always been exemplary in cancer studies. Signaling cascade of MAPK, comprising JNK, p38 and ERK1/2, are involved in cellular survival as well as death. Most of the times, JNK and p38 initiates cascade leading towards cell death while ERK1/2 promotes cell survival (Sui et al., 2014; Ren et al., 2017). Previous reports indicate that PSD-A increased phosphorylation of p38 and JNK leading towards apoptotic death in A549 lung cancer cells (Maryam et al., 2018). Interestingly, JNK, due to its dual role in death and survival pathway, induces autophagic events due to numerous stimulatory actions including ER stress (Ogata et al., 2006). Researchers have revealed that ROS-dependent JNK activation can trigger both apoptosis and autophagy at same time (Wong et al., 2010). More importantly, dopaminergic-specific neurotoxin MPP^+^ induced autophagy has been reported to be aroused by oxidative stress, mediated by JNK activation and inactivated by AKT/mTOR signaling pathway (Rodriguez-Blanco et al., 2012). Therefore, we were interested in intricating the role of MAPK and mTOR/AKT signaling in PSD-A induced apoptosis and autophagy. In our study, PSD-A activated JNK and p38 very early in its exposure while inhibited mTOR and AKT phosphorylation along with inhibition of ERK1/2 phosphorylation in 24 h treatment. We investigated the possible role of JNK and p38 in apoptosis and autophagy by using their pharmacological inhibitors. In our study, SP600125 (JNK inhibitor) successfully inhibited JNK activation, improved PSD-A induced PARP cleavage and reduced conversion of LC3-I to LC3-II suggesting that PSD-A induces JNK activation leading to autophagy. Moreover, pretreatment of SP600125 facilitated inhibition of STAT3 activation suggesting its role in stimulating apoptotic rate *via* inhibiting JNK-dependent autophagy.

The role of p38 MAPK in mediation of apoptosis and autophagy in response to the chemotherapeutics has become an intensive field in cancer studies. In both, apoptosis and autophagy, p38 MAPK plays dual role i.e. positive as well negative regulator (Sui et al., 2014). Earlier, role of p38 MAPK and SB203580 in apoptosis and autophagy has been well documented in SW620 colorectal cancer (Xue et al., 2015). In our data, SB203580 inhibited p38 phosphorylation, improved PARP cleavage in MCF-7 cells and elevated rate of LC3-I conversion in to LC3-II.

**Figure 8:**
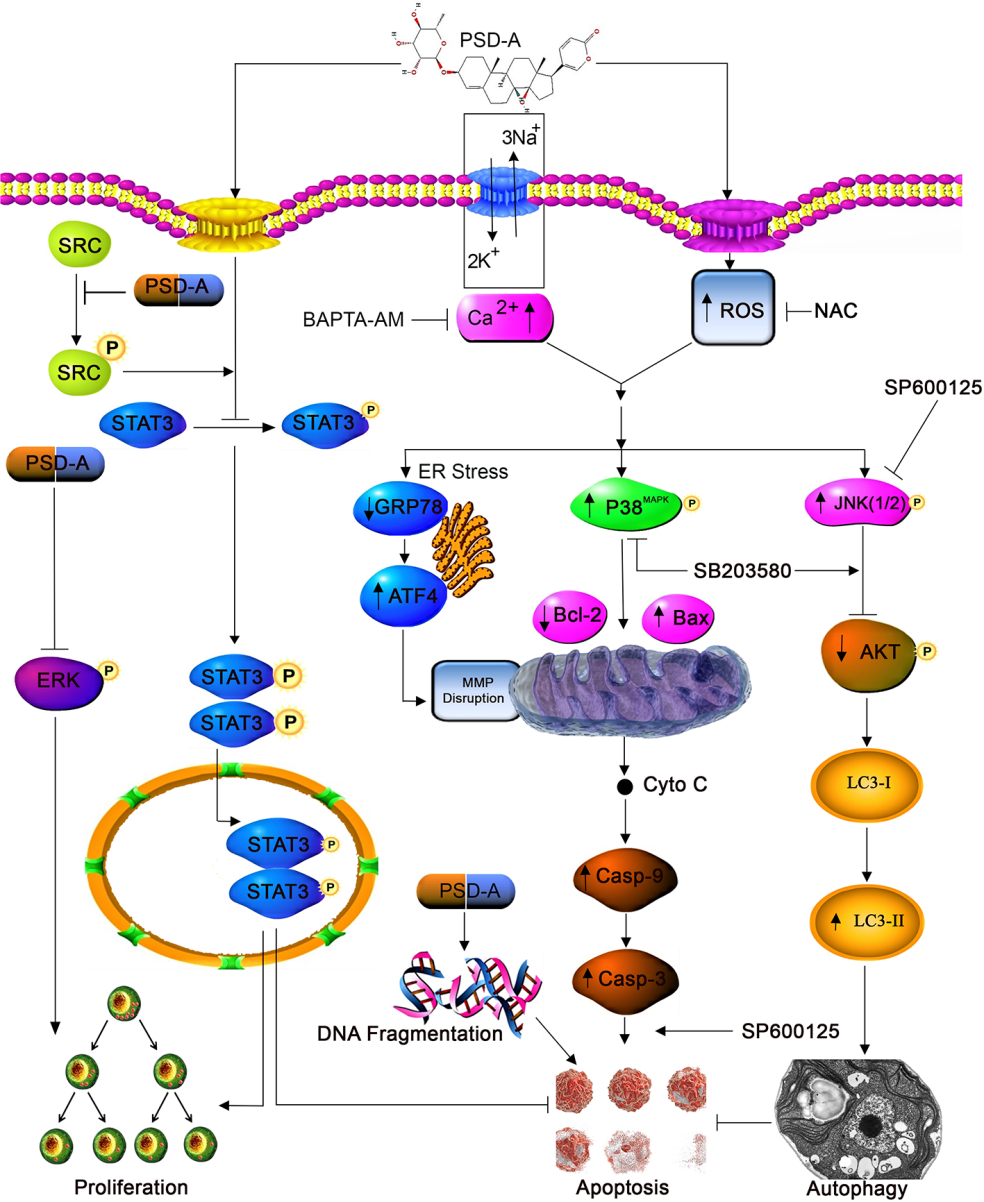
A schematic model for molecular mechanism of PSD-A-induced anticancer activity in MCF-7 and MDA-MB-231breast carcinoma cells.

In conclusion, we have evidenced the anticancer activity of PSD-A along with the detailed underlying mechanism in both ER-positive and ER-negative breast cancer cells. PSD-A induced mitochondrial apoptotic cell death *via* ROS generation and intracellular Ca^+2^ accumulation. Moreover, PSD-A inhibited cytoprotective ER stress chaperone GRP78. Also, the comprehensive molecular mechanism of inhibition of STAT3 activation by PSD-A has been disclosed for the first time in breast cancer cells. We have, firstly, evaluated the role of PSD-A in initiation of JNK-dependent autophagic events *via* mTOR/AKT pathway. Our data reveal that the blocking of autophagy improves apoptotic death *via* involving STAT3 but further investigations are still needed to get a complete understanding of cross talk between apoptosis and autophagy. Also unravelling the limitation of *in vivo* approach in current mechanistic study would give a better understanding to step forward for pre-clinical tactics. A schematic model for the molecular mechanism of PSD-A has been shown in **Figure 8**. Taken together, our data suggest that PSD-A not only can become potential anticancer therapeutics for breast cancer but can also augments anti-tumor activity of available drugs due to its multi-facet anticancer properties that need to be explored further.

## Data Availability Statement

All datasets generated for this study are included in the article/supplementary material.

## Author Contributions

MS proposed conceptualization, optimized protocols for the study, conducted the experiments and prepared original first draft of manuscript. TM and MK assisted in conceptualization, data analysis, provided financial assistance for project administration, edited manuscript and supervised the study. SA, GA, MA and MN undertook computational and statistical analysis while MA aided optimizing protocols. HZ, LJ and SD offered technical assistance.

## Funding 

This research work was supported by research grants from National Natural Science Foundation of China (Grant No. 81650110526) and Pandeng Scholarship Program of Liaoning Province.

## Conflict of Interest

The authors declare that the research was conducted in the absence of any commercial or financial relationships that could be construed as a potential conflict of interest.
